# Multi-task learning approach for utilizing temporal relations in natural language understanding tasks

**DOI:** 10.1038/s41598-023-35009-7

**Published:** 2023-05-26

**Authors:** Chae-Gyun Lim, Young-Seob Jeong, Ho-Jin Choi

**Affiliations:** 1 School of Computing, KAIST, Daejeon, 34141 South Korea; 2 Department of Computer Engineering, Chungbuk National University, Cheongju, 28644 South Korea

**Keywords:** Computer science, Information technology

## Abstract

Various studies have been conducted on multi-task learning techniques in natural language understanding (NLU), which build a model capable of processing multiple tasks and providing generalized performance. Most documents written in natural languages contain time-related information. It is essential to recognize such information accurately and utilize it to understand the context and overall content of a document while performing NLU tasks. In this study, we propose a multi-task learning technique that includes a temporal relation extraction task in the training process of NLU tasks such that the trained model can utilize temporal context information from the input sentences. To utilize the characteristics of multi-task learning, an additional task that extracts temporal relations from given sentences was designed, and the multi-task model was configured to learn in combination with the existing NLU tasks on Korean and English datasets. Performance differences were analyzed by combining NLU tasks to extract temporal relations. The accuracy of the single task for temporal relation extraction is 57.8 and 45.1 for Korean and English, respectively, and improves up to 64.2 and 48.7 when combined with other NLU tasks. The experimental results confirm that extracting temporal relations can improve its performance when combined with other NLU tasks in multi-task learning, compared to dealing with it individually. Also, because of the differences in linguistic characteristics between Korean and English, there are different task combinations that positively affect extracting the temporal relations.

## Introduction

With recent rapid technological developments in various fields, numerous studies have attempted to achieve natural language understanding (NLU). The NLU field is not limited to the tasks of named entity recognition, dependency analysis, and relationship extraction, but includes research that more effectively reflects the context of natural language using a model that handles multiple tasks simultaneously. Multi-task learning (MTL) has recently drawn attention because it better generalizes a model for understanding the context of given documents^1^. Benchmark datasets, such as GLUE^2^ and KLUE^3^, and some studies on MTL (e.g., MT-DNN^1^ and decaNLP^4^) have exhibited the generalization power of MTL.

**Figure 1:**
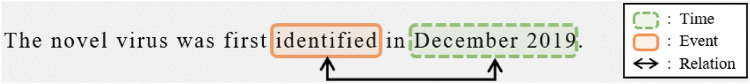
Example of temporal information. There is an example sentence “The novel virus was first identified in December 2019.” In this sentence, the verb ‘identified’ is annotated as an EVENT entity, and the phrase ‘December 2019’ is annotated as a TIME entity. Thus, two entities have a temporal relationship that can be annotated as a single TLINK entity.

**Figure 2:**
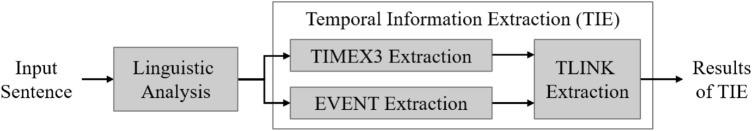
Workflow of temporal information extraction. When an input sentence is provided, a process of linguistic analysis is applied as preprocessing. timex3 and event extraction precede tlink extraction.

Among the tasks in the NLU field, information extraction of temporal expressions is important because sentences contain temporal expressions (e.g., today, next year, and October 3rd), and temporal information is necessary to better understand the context of natural language sentences and develop various applications, such as conversational agents or Q&A systems^5^. Specifically, temporal information extraction comprises three subtasks: timex3 extraction for temporal expressions, event extraction for event expressions, and tlink extraction for temporal relationships. Figure 1 shows an example, where the verb ‘identified’ is an event (i.e., event tag), the phrase ‘December 2019’ is a temporal expression (i.e., timex3 tag), and there will be a temporal relation (i.e., tlink tag) between the event tag and timex3 tag. There have been shared tasks such as TempEval-3^6^, Clinical TempEval^7–9^, and i2b2 Challenge on Temporal Relations^10^. Figure 2 shows the method for performing temporal information extraction. The tlink extraction is more difficult than the two other subtasks as it needs to consider a broader context to obtain the relationship between different tags; moreover, poorly extracted timex3 and event will cause poorly extracted tlink tags.

In this study, we propose a new MTL approach that involves several tasks for better tlink extraction. We designed a new task definition for tlink extraction, TLINK-C, which has the same input as other tasks, such as semantic similarity (STS), natural language inference (NLI), and named entity recognition (NER). The difference between TLINK-C and tlink is that the TLINK-C extraction takes a raw sentence as input and gives a type of temporal relation, whereas the tlink extraction usually takes a pair of timex3 and event tags and gives a type of temporal relation between them. We prepared an annotated dataset for the TLINK-C extraction task by parsing and rearranging the existing datasets. We investigated different combinations of tasks by experiments on datasets of two languages (e.g., Korean and English), and determined the best way to improve the performance on the TLINK-C task. In our experiments on the TLINK-C task, the individual task achieves an accuracy of 57.8 on Korean and 45.1 on English datasets. When TLINK-C is combined with other NLU tasks, it improves up to 64.2 for Korean and 48.7 for English, with the most significant task combinations varying by language. We also examined the reasons for the experimental results from a linguistic perspective.

The contributions of this study are summarized as follows:

- The extraction task of temporal relations, TLINK-C, is designed as an end-to-end task applicable to the MTL approach.
- The MTL approach is presented that can be trained on existing NLU tasks and TLINK-C in parallel, and its performance is measured on various task combinations with Korean and English languages.

The remainder of this study is organized as follows. “Related works” section introduces the MTL-based techniques and research on temporal information extraction. “Proposed approach” section describes the proposed approach for the TLINK-C extraction. “Experiments” section demonstrates the performance of various combinations of target tasks through experimental results. Finally, “Conclusion” section concludes the paper.

## Related works

Recently, deep learning (DL) techniques become preferred to other machine learning techniques. This may be mainly because the DL technique does not require significant human effort for feature definition to obtain better results (e.g., accuracy). In addition, studies have been conducted on temporal information extraction using deep learning models. Meng et al.^11^ used long short-term memory (LSTM)^12^ to discover temporal relationships within a given text by tracking the shortest path of grammatical relationships in dependency parsing trees. They achieved 84.4, 83.0, and 52.0% of F1 scores for the timex3, event, and tlink extraction tasks, respectively. Laparra et al.^13^ employed character-level gated recurrent units (GRU)^14^ to extract temporal expressions and achieved a 78.4% F1 score for time entity identification (e.g., May 2015 and October 23rd). Kreimeyer et al.^15^ summarized previous studies on information extraction in the clinical domain and reported that temporal information extraction can improve performance. Temporal expressions frequently appear not only in the clinical domain but also in many other domains. Therefore, even though previous studies achieved good performance, we argue that we still need to elaborate more on temporal information extraction, especially on tlink extraction because its performance has been significantly lower than that of the others (e.g., timex3 and event).

**Figure 3:**
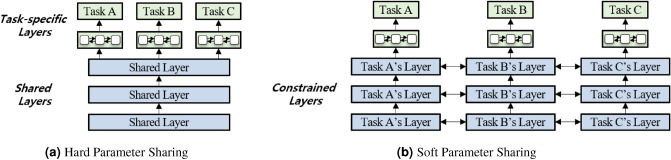
Two types of MTL methods to learn the hidden layers^16^.

The multi-task learning (MTL) approach has been applied to numerous tasks. This approach forces a model to address several different tasks simultaneously, and may allow the incorporation of the underlying patterns of different tasks such that the model eventually works better for the tasks. There are mainly two ways (e.g., *hard parameter sharing* and *soft parameter sharing*) of architectures of MTL models^16^, and Fig. 3 illustrates these ways when a multi-layer perceptron (MLP) is utilized as a model. Soft parameter sharing allows a model to learn the parameters for each task, and it may contain constrained layers to make the parameters of the different tasks similar. Hard parameter sharing involves learning the weights of shared hidden layers for different tasks; it also has some task-specific layers. Both methods allow the model to incorporate learned patterns of different tasks; thus, the model provides better results. For example, Liu et al.^1^ proposed an MT-DNN model that performs several NLU tasks, such as single-sentence classification, pairwise text classification, text similarity scoring, and correlation ranking. McCann et al.^4^ proposed decaNLP and built a model for ten different tasks based on a question-and-answer format. Also, Fei et al.^17^ developed a structure-aware generative language model, which can cover heterogeneous NLU tasks as a unified model by reducing them into three different prototypes—i.e., span extraction, pair extraction, and hyper-pair extraction tasks. These studies demonstrated that the MTL approach has potential as it allows the model to better understand the tasks.

Although the MTL approach has the potential for performance improvement, it does not always yield better results. Therefore, previous studies have investigated different combinations of tasks to obtain better results. Changpinyo et al.^18^ empirically compared the performance using combinations of multiple tasks in the sequence tagging problem. In their study, there are 11 tasks—universal and English-specific POS tagging (UPOS and XPOS), syntactic chunking (CHUNK), named entity recognition (NER), multi-word expression identification (MWE), supersense tagging (SUPSENSE), supersense (SEM) and semantic trait (SEMTR) tagging, sentence compression (COM), frame target identification (FRAME), hyperlink detection (HYP). The experimental results showed that combining the UPOS, XPOS, or CHUNK tasks improved the performance of other tasks, whereas combining the COM, FRAME, or HYP tasks reduced the performance of other tasks. This implies that tasks approached by the token level in given sentences, such as POS or chunking in sequence tagging, positively affect the model’s performance. Sanh et al.^19^ presented the experimental results of MTL by configuring four tasks: NER, entity mention detection (EMD), relation extraction (RE), and coreference resolution (CR). Considering the performance of RE representatively, their model’s F1-score improved to 61.30 after multi-task learning compared to 55.99 in single-task learning.

As shown in previous studies, MTL methods can significantly improve model performance. However, the combination of tasks should be considered when precisely examining the relationship or influence between target NLU tasks^20^. Zhang et al.^21^ explained the influence affected on performance when applying MTL methods to 40 datasets, including GLUE and other benchmarks. Their experimental results showed that performance improved competitively when learning related tasks with high correlations or using more tasks. Therefore, it is significant to explore tasks that can have a positive or negative impact on a particular target task. In this study, we investigate different combinations of the MTL approach for TLINK-C extraction and discuss the experimental results.

## Proposed approach

**Figure 4:**
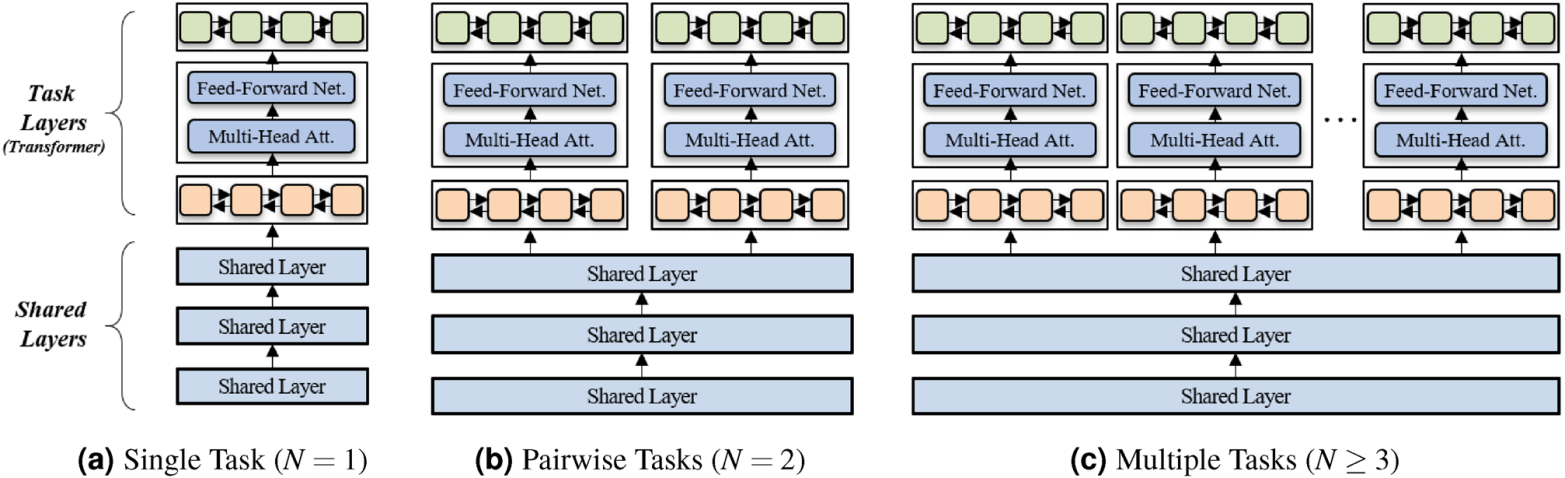
MTL architecture of different combinations of tasks, where *N* indicates the number of tasks.

We develop a model specializing in the temporal relation classification (TLINK-C) task, and assume that the MTL approach has the potential to contribute to performance improvements. As shown in Fig. 4, we designed deep neural networks with the hard parameter sharing strategy in which the MTL model has some task-specific layers and shared layers, which is effective in improving prediction results as well as reducing storage costs. As the MTL approach does not always yield better performance, we investigated different combinations of NLU tasks by varying the number of tasks *N*.

### Temporal relation classification task

In previous studies, temporal information extraction has been divided into three sub-tasks: time expression (timex3), event expression (event), and temporal relation (tlink), as described in the TempEval^6^ shared task (Fig. 2). We address the temporal relation extraction subtask, where the tlink tag is the relationship between two other tags (timex3 and event). From the perspective of various NLU tasks, these relationships may have a significant effect on understanding the context of the given texts in terms of temporal reasoning or causal inference. In traditional studies, timex3 and event tags are extracted, and the relationships between them are connected. Because this two-step extraction task has not been well suited to performing end-to-end tasks using pre-trained language models in recent years, we form a classification task for grasping temporal relations based on the given input text. In this study, we assumed that the timex3 and event tags are closely related to other tasks. For example, the timex3 and event might be considered as special types of named entities (NEs), and the event is mainly a verbal phrase (e.g., ‘eat,’ and ‘run’), thus it should be related to an NLI task that involves the semantic meaning of eventual phrases (e.g., ‘eat’ versus ‘starve’). Based on this assumption, we employ the MTL approach, which benefits from other related tasks.

**Figure 5:**
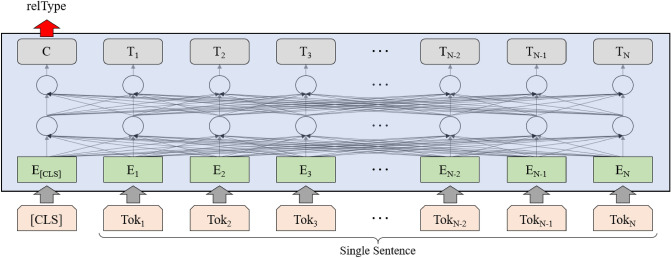
Task design for temporal relation classification (TLINK-C) as a single sentence classification. We separate a given sentence into word tokens as our task’s input. The first token is a special token ‘[*CLS*]’ to predict a class label. When our task is trained, the latent weight value corresponding to the special token is used to predict a temporal relation type.

We define the TLINK-C extraction task as sentence classification over multiple classes of temporal relations (e.g., begins, ends, and during); in particular, it is designed to classify the temporal relation (i.e., relation type) that the model grasps from a given sentence. Assuming that we utilize the architecture of bidirectional encoder representations from transformers (BERT)^22^, the input sequence S = $\{[CLS], t_1, t_2, ..., t_N\}$ is fed into the model, and ‘relType’ (i.e., relation type) will be obtained from a softmax function at the left-most output of the top layer, as shown in Fig. 5. If the input sentence is “I bought a car, December 2019,” then the relation type will be ‘includes’ between timex3 (December 2019) and event (bought). The input of the TLINK-C task is a token sequence, which is the same as that of other related tasks (e.g., NER and NLI), allowing us to easily adopt the MTL approach with the related tasks.

**Figure 6:**
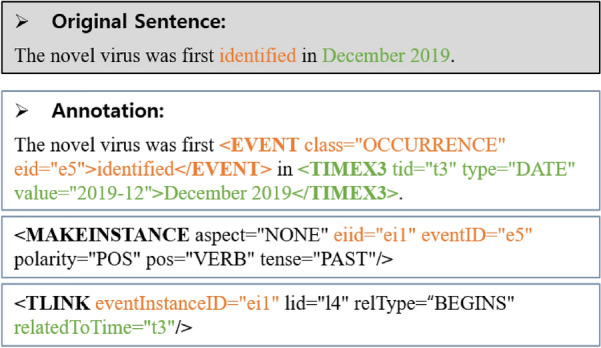
Example of annotated temporal information. TIMEX3 and EVENT expressions are tagged with specific markup notations, and a TLINK is individually assigned by linking the relationship between them.

We built a new dataset for the TLINK-C task by parsing and rearranging two previous datasets, Korean TimeBank^23^ and i2b2 Challenge^10^. Figure 6 shows an annotated example in an in-line fashion that follows the previous scheme of temporal information extraction; timex3 and event expressions are tagged with specific mark-up notations, makeinstance plays a role of an instance of the event tag, and tlink tag links two other tags. By parsing the annotated data, we found the corresponding sentence of the tlink tag and removed the markup notations of the timex3 and event tags. The obtained original sentence was paired with the relType (relation type) of the tlink tag, where the attribute relType may have a value among eight relation classes: before, after, includes, during, begins, ends, identity, and simultaneous.

### NLU tasks for MTL approach

In this study, we aimed to observe the positive or negative changes that occur due to the correlation among tasks when learning the TLINK-C task along with existing NLU tasks. We also determined whether the interactions between the TLINK-C task and other tasks differ by changing the linguistic characteristics and potential features. We specifically targeted particular tasks commonly included in Korean and English benchmarks to utilize existing pre-trained language models. Based on the assumption that timex3 and event are related to NLU tasks, we selected three tasks for the MTL approach: semantic similarity, natural language inference, and named entity recognition. These tasks were included in GLUE^2^ and KLUE^3^, which provided Korean datasets for several NLU tasks.

- *Semantic similarity (STS):* This task evaluates two input sentences that are semantically similar to the training model. Performance was measured using Pearson-Spearman correlation coefficients.
- *Natural language inference (NLI):* Considering a particular premise and hypothesis sentence, the training model classified hypotheses into entailment, contraction, and neutral. The performance was measured by the accuracy of the classification results.
- *Named entity recognition (NER):* The training model discovers the range of named entities in a given sentence and classifies them into specific types. The performance was measured by the precision and recall of the classification results. There are six types of entity names used in KLUE-NER: person (PS), location (LC), organization (OG), date (DT), time (TI), and quantity (QT). Each data point was tagged in the form of a begin-inside-outside (BIO) in character unit.

We tested different combinations of the above three tasks along with the TLINK-C task. During the training of the model in an MTL manner, the model may learn promising patterns from other tasks such that it can improve its performance on the TLINK-C task.

## Experiments

### Experimental setting

**Table 1:** Summary of dataset size compared to KLUE and GLUE tasks.

Language	Task	# Train	# Valid	# Test
Korean	STS	9335	519	2333
	NLI	19,999	3000	4999
	NER	16,807	5000	4201
	TLINK-C	1804	450	250
English	STS	5749	1500	1379
	NLI	549,367	9842	9824
	NER	14,041	3250	3453
	TLINK-C	14,056	4016	2009

In this study, multi-task learning was constructed based on the *MTDNN-base-uncased* model^1^ that had been trained from BERT^22^, and the learning performance was measured by combining four learning target tasks: STS, NLI, NER, and TLINK-C. Table 1 summarizes the amount of data for each task compared to the KLUE and GLUE benchmarks. Only training and validation data exist in dataset v1.1, which is provided in the KLUE benchmark. Thus, we divided 20% of the training data and used it as the test data. TLINK-C datasets of Korean and English are randomly sampled from the Korean TimeBank^23^ corpus and i2b2 Challenge on Temporal Relations^10^. In the cases of NLI and TLINK-C, the amount of Korean data is significantly smaller than that of English data. To conduct a fair experiment, we undersampled the amount of English data to match the quantity on the small side of the Korean data. The training parameters are $batch\_size=8$, $training\_epoch=5$, $optimizer=$“adamx”, $learning\_rate=2e-5$, and $gradient\_accumulation$ $\_step=4$.

**Table 2:** Comparison on the performance among the target task combinations for Korean dataset. Significant values are in bold.

Tasks		STS (Pearson/Spearman)	NLI (accuracy)	NER (precision/recall)	TLINK-C (accuracy)
Each single task		65.212/64.752	54.400	0.4854/0.5179	57.778
Pairwise	STS + NLI	**68.459/67.918**	55.667	−	−
	STS + NER	67.347/67.364	−	0.3946/0.3978	−
	STS + TLINK-C	65.977/65.918	−	−	50.667
	NLI + NER	−	**56.833**	0.4108/0.4134	−
	NLI + TLINK-C	−	54.900	−	54.444
	NER + TLINK-C	−	−	**0.4685/0.5030**	**63.111**
Multiple	STS + NLI + NER	**70.655/71.062**	56.467	0.3629/0.3468	−
	STS + NLI + TLINK-C	69.131/68.571	55.400	−	52.667
	STS + NER + TLINK-C	68.198/68.359	−	0.3706/0.3675	63.556
	NLI + NER + TLINK-C	−	**57.167**	**0.3950/0.3947**	**64.222**
	STS + NLI + NER + TLINK-C	69.404/69.221	56.900	0.3632/0.3470	62.667

**Table 3:** Comparison on the performance among the target task combinations for English dataset. Significant values are in bold.

Tasks		STS (Pearson/Spearman)	NLI (accuracy)	NER (precision/recall)	TLINK-C (accuracy)
Each single task		89.741/89.688	86.200	0.9862/0.9851	45.111
Pairwise	STS + NLI	89.300/89.139	86.367	−	−
	STS + NER	89.631/89.617	−	0.9839/0.9822	−
	STS + TLINK-C	**89.829/89.654**	−	−	48.000
	NLI + NER	−	**86.433**	0.9825/0.9809	−
	NLI + TLINK-C	−	86.267	−	**48.667**
	NER + TLINK-C	−	−	**0.9861/0.9850**	48.000
Multiple	STS + NLI + NER	89.439/89.243	**86.967**	0.9823/0.9807	−
	STS + NLI + TLINK-C	89.533/89.338	86.100	−	**48.667**
	STS + NER + TLINK-C	**89.849/89.764**	−	**0.9840/0.9827**	48.222
	NLI + NER + TLINK-C	−	85.833	0.9831/0.9816	48.000
	STS + NLI + NER + TLINK-C	89.376/89.210	85.833	0.9823/0.9810	48.000

### Results

Tables 2 and 3 present the results of comparing the performance according to task combination while changing the number of learning target tasks *N* on the Korean and English benchmarks, respectively. The groups were divided according to a single task, pairwise task combination, or multi-task combination. The result showing the highest task performance in the group are highlighted in bold.

First, we present the experimental results for the Korean benchmarks (Table 2). Overall, in the case of the STS and NLI tasks, the performance improved when learning along with other tasks, whereas the NER task showed a tendency to deteriorate as more tasks were learned together. In the TLINK-C task, there were mixed cases in which the performance improved or declined depending on the task combination. Notably, the learning performance of TLINK-C is lowered to 50.667 or 54.444 for pairwise cases with STS or NLI tasks, respectively, compared with the individual performance of 57.778. However, when the TLINK-C task was combined with the NER task, the performance improved to at least 62.667 or higher in all cases.

Next, we describe the experimental results for the English benchmarks (Table 3). This table shows the results for the undersampled dataset with the same number as the KLUE dataset; however, we confirmed that this result has a similar tendency without much difference from the other experiments using all the data. Similar to the results of the Korean experiment, NLI improved performance in multi-task combinations, and NER degraded in all combinations. This is because NER identifies each entity’s type at the token level within a given sentence, and thus the granularity of the target task is different. However, some results differ from those of the Korean experiment. STS improved performance only when combined with TLINK-C. This differs from the Korean results, which showed improved performance for every task combination. TLINK-C improved the performance when combined with NLI to 48.667 from an individual of 45.111, unlike the performance degradation observed in the Korean results. Although the combination “NLI+NER+TLINK-C” showed the highest performance in the Korean case, we can observe that TLINK-C is conversely degraded when combined with “NLI+NER” of 48.000, which is the same as the pairwise case of only NER in English results. Considering the NLI and NER tasks simultaneously in English, there is a possibility of potential factors conflicting with temporal context. Based on these experimental results, we can observe that the performance of NLU tasks can have different influences depending on linguistic characteristics. In addition, as in comparing Korean and English, even the same NLU task could have different effects depending on the type of language.

**Figure 7:**
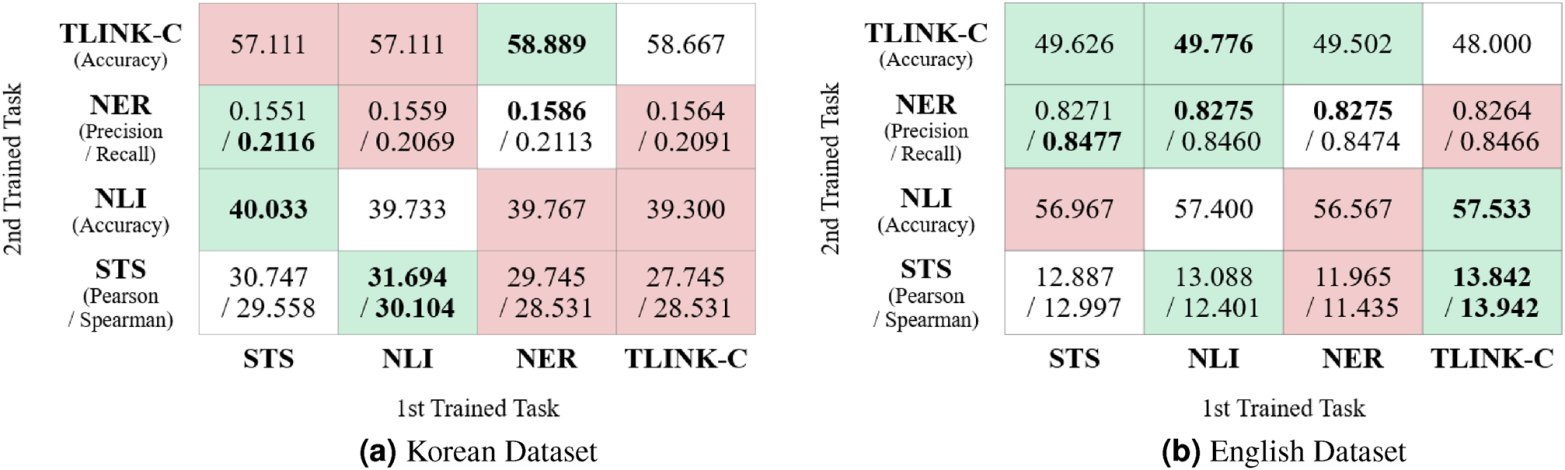
Performance of the transfer learning for pairwise task combinations instead of applying the MTL model. It shows the results of learning the 2nd trained task (i.e, target task) in the vertical axis after learning the 1st trained task in the horizontal axis first using a pre-trained model. The diagonal values indicate baseline performance for each individual task without transfer learning. The result showing the highest task performance are highlighted in bold. In addition, the background color is represented in green if the performance of transfer learning is better than the baseline and in red otherwise.

To confirm the performance with transfer learning rather than the MTL technique, we conducted additional experiments on pairwise tasks for Korean and English datasets. Figure 7 shows the performance comparison of pairwise tasks applying the transfer learning approach based on the pre-trained *BERT-base-uncased* model. Unlike the performance of Tables 2 and 3 described above is obtained from the MTL approach, this result of the transfer learning shows the worse performance. Regarding Korean tasks in Fig. 7a, we can see that NLI and STS tasks have a positive correlation with each other, improving the performance of the target task by transfer learning. In contrast, in the case of the NER task, learning STS first improved its performance, whereas learning NLI first degraded. TLINK-C task improved its performance only if NER was learned first. On the other hand, regarding English tasks in Fig. 7b, the performance of all the tasks improved when learning the NLI task first. Learning the TLINK-C task first improved the performance of NLI and STS, but the performance of NER degraded. Also, the performance of TLINK-C always improved after any other task was learned.

The increase or decrease in performance seems to be changed depending on the linguistic nature of Korean and English tasks. Although we can see that the trend of performance changes is similar to that of the MTL approach’s results to the pairwise tasks, the performance of the target task varies depending on the training order in which the transfer learning is performed. In the existing study on the analysis of the performance differences of the NLU tasks when applying MTL and transfer learning approaches, it is revealed that the model’s capability to do tasks learned in the past is reduced if the model learns other target tasks due to *catastrophic forgetting* problem^24^. Considering the situation that attempts to train a desired target task after learning a supporting task first, the hidden features obtained from the supporting task will be forgotten in the case of transfer learning, while the MTL model continued to utilize the supporting task during the training process. From this perspective, we believe that the MTL approach is a better way to effectively grasp the context of temporal information among NLU tasks than using transfer learning.

**Table 4:**
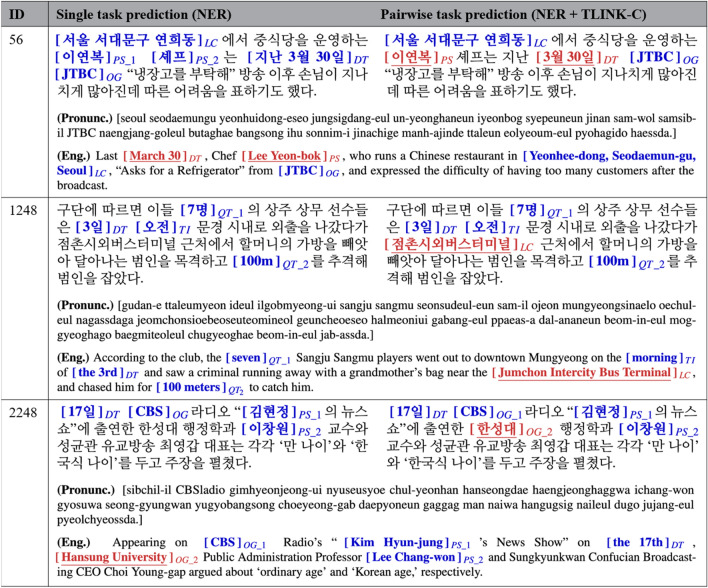
Examples of NER task’s predictions in the Korean dataset.

Meanwhile, we also present examples of a case study applying multi-task learning to traditional NLU tasks—i.e., NER and NLI in this study—alongside the TLINK-C task. In our previous experiments, we discovered favorable task combinations that have positive effects on capturing temporal relations according to the Korean and English datasets. For Korean, it was better to learn the TLINK-C and NER tasks among the pairwise combinations; for English, the NLI task was appropriate to pair it. It was better to learn TLINK-C with NER together for Korean; NLI for English. Table 4 shows the predicted results in several Korean cases when the NER task is trained individually compared to the predictions when the NER and TLINK-C tasks are trained in a pair. Here, ID means a unique instance identifier in the test data, and it is represented by wrapping named entities in square brackets for each given Korean sentence. At the bottom of each row, we indicate the pronunciation of the Korean sentence as it is read, along with the English translation. Named entities emphasized with underlining mean the predictions that were incorrect in the single task’s predictions but have changed and been correct when trained on the pairwise task combination. In the first case, the single task prediction determines the spans for ‘이연복 (Lee Yeon-bok)’ and ‘셰프 (Chef)’ as separate PS entities, though it should only predict the parts corresponding to people’s names. Also, the whole span for ‘지난 3월 30일 (Last March 30)’ is determined as a DT entity, but the correct answer should only predict the exact boundary of the date, not including modifiers. In contrast, when trained in a pair with the TLINK-C task, it predicts these entities accurately because it can reflect the relational information between the entities in the given sentence. Similarly, in the other cases, we can observe that pairwise task predictions correctly determine ‘점촌시외버스터미널 (Jumchon Intercity Bus Terminal)’ as an LC entity and ‘한성대 (Hansung University)’ as an OG entity. Table 5 shows the predicted results for the NLI task in several English cases. These examples present several cases where the single task predictions were incorrect, but the pairwise task predictions with TLINK-C were correct after applying the MTL approach. Since the NLI task is intended to determine the semantic information between a premise and a hypothesis, learning the TLINK-C task together was able to reflect the latent relational information within given sentences to obtain the correct result. As a result of these experiments, we believe that this study on utilizing temporal contexts with the MTL approach has the potential capability to support positive influences on NLU tasks and improve their performances.

**Table 5:** Examples of NLI task’s predictions in the English dataset.

ID	Premise	Hypothesis	Gold label	Single task prediction (NLI)	Pairwise task prediction (NLI + TLINK-C)
1073	A man in a mask in the back of a service vehicle	A robber is in a police car	Neutral	Contradiction	Neutral
1821	A band performing at a local bar or club	The band is playing music at a large venue	Contradiction	Entailment	Contradiction
4784	The man is outside on the beach walking his dog	There is sand in the ground	Entailment	Neutral	Entailment

## Conclusion

In this study, we proposed the multi-task learning approach that adds the temporal relation extraction task to the training process of NLU tasks such that we can apply temporal context from natural language text. This task of extracting temporal relations was designed individually to utilize the characteristics of multi-task learning, and our model was configured to learn in combination with existing NLU tasks on Korean and English benchmarks. In the experiment, various combinations of target tasks and their performance differences were compared to the case of using only individual NLU tasks to examine the effect of additional contextual information on temporal relations. Generally, the performance of the temporal relation task decreased when it was pairwise combined with the STS or NLI task in the Korean results, whereas it improved in the English results. By contrast, the performance improved in all cases when combined with the NER task.

In future work, we plan to select additional NLU tasks for comparative experiments and analyze the influencing factors that may occur in target tasks of different natures by inspecting all possible combinations of time-related NLU tasks.

## Data Availability

The datasets provided by GLUE (https://gluebenchmark.com/tasks) and KLUE (https://klue-benchmark.com/tasks) benchmarks are publicly available. Another dataset, Korean TimeBank (https://lremap.elra.info/?languages=Korean &q=TimeBank+v2.0), is not publicly available due to the fact that they constitute an excerpt of research in progress but are available from the corresponding author on reasonable request.
